# Supplementary figures and images for: Aboveground insect herbivory increases plant competitive asymmetry, while belowground herbivory mitigates the effect
Source: PeerJ. 2016 Apr 4;4:e1867. doi: 10.7717/peerj.1867 (PMC4824911; doi:10.7717/peerj.1867)

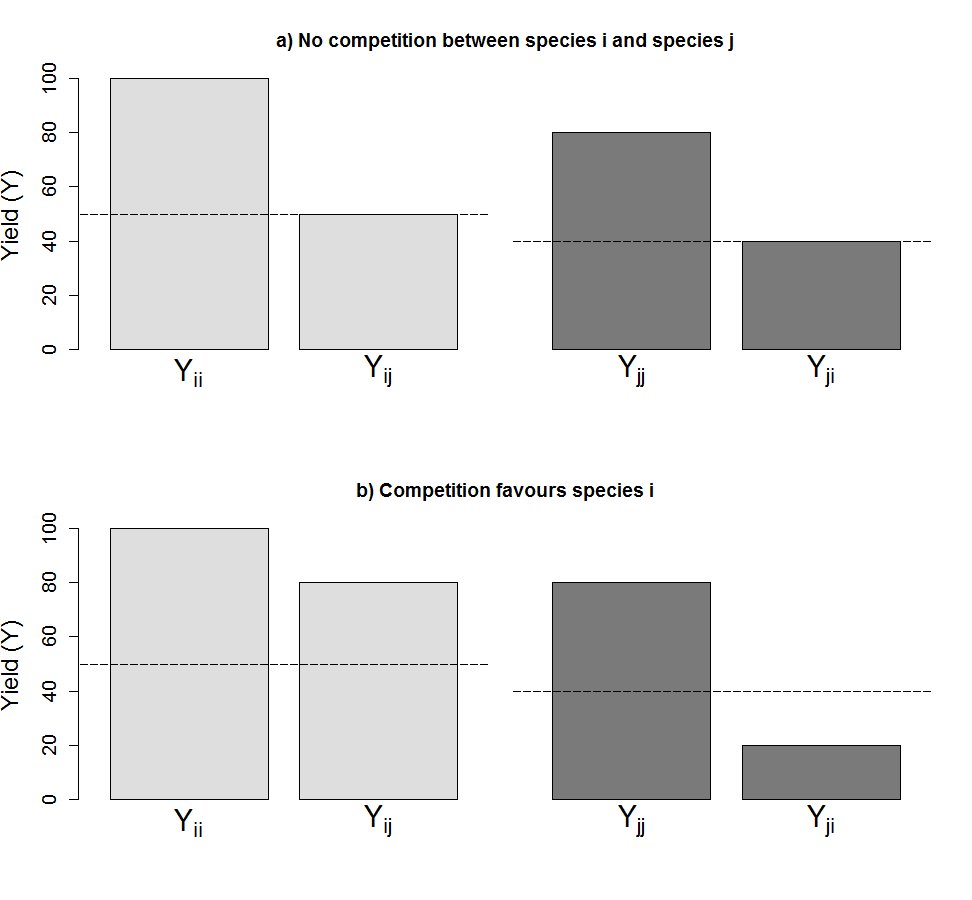

Supplement: Figure S1 — Species i and j are grown in monoculture and in mixture, and the yields (Y) of the respective cultures are measured. The dashed line in each case shows the outcome if performance in monoculture and in mixture are the same (RY = 0.5), i.e., if the species is unaffected by the presence of the other species. In (A) species j produces less biomass than species i does, both in monoculture and in mixture. However, the yield of species j in monoculture is proportional to that in mixture. For species i, RY = 50∕100 = 0.5. For species j, RY = 40∕80 = 0.5. Aggressivity of the competitive interaction in this case is A = 0.5–0.5 = 0, i.e., there is no competition between the two species. In (B) species j still produces less than species i does in monoculture, but in this case the yield is proportionally less for species j in mixture. Here, RYi = 80∕100 = 0.8, while RYj = 20∕80 = 0.25. Aggressivity of the competitive interaction is A = 0.8–0.25 = 0.55. [file peerj-04-1867-s001.jpg]

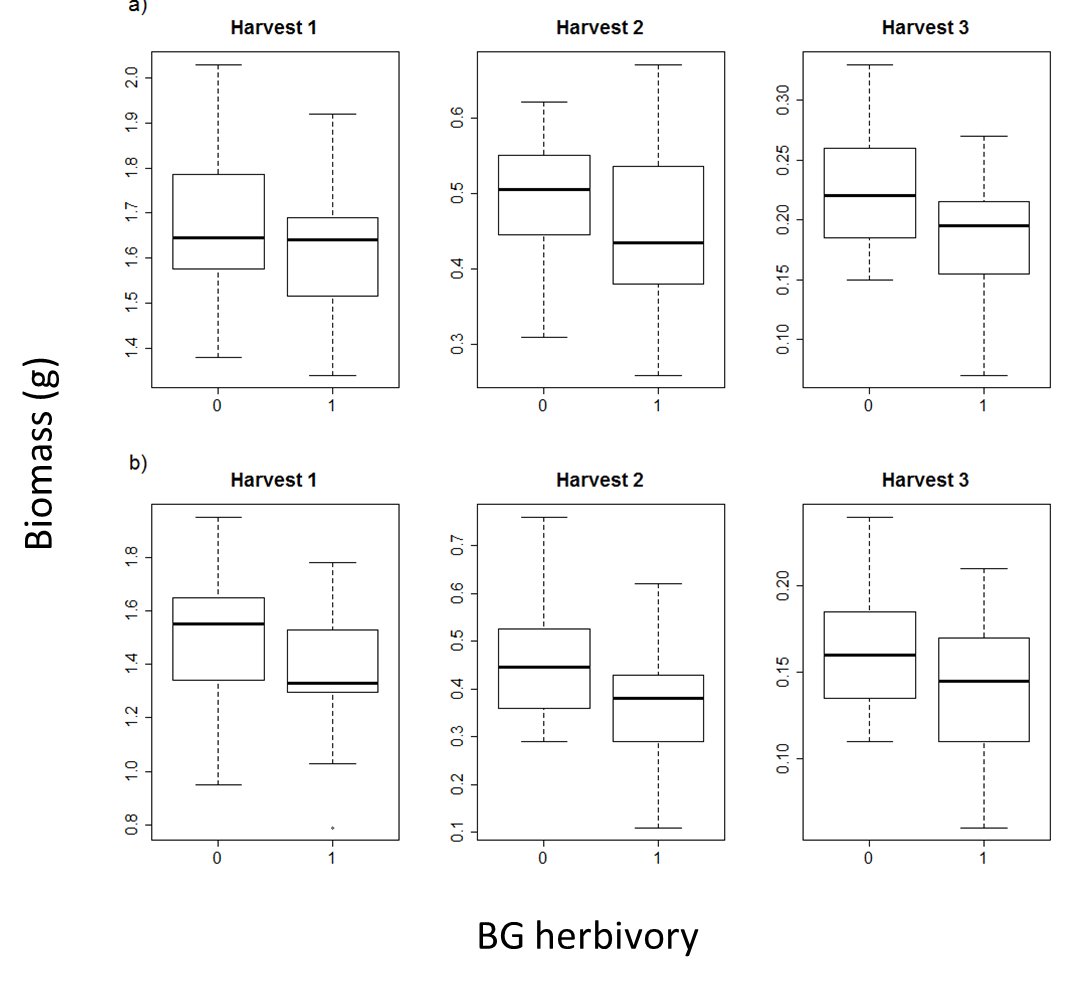

Supplement: Figure S2 — The plots show aboveground biomass for each species at each harvest, with and without BG herbivory, and under contrasting nitrogen levels. Although BG herbivory appears to lower aboveground biomass production, the effect is not statistically detectable. [file peerj-04-1867-s002.jpg]
