# Supplementary material for: Aboveground insect herbivory increases plant competitive asymmetry, while belowground herbivory mitigates the effect
Source: PeerJ. 2016 Apr 4;4:e1867. doi: 10.7717/peerj.1867 (PMC4824911; doi:10.7717/peerj.1867)
Supplement: Table S3 [file peerj-04-1867-s003.docx]

|  | **Aggressivity** | | **Relative Yield** | | | |
| --- | --- | --- | --- | --- | --- | --- |
| **Treatment** |  | | *Dactylis glomerata* | | *Festuca rubra* | |
|  | ***F-*value** | ***P*-value** | ***F*-value** | ***P*-value** | ***F*-value** | ***P*-value** |
| **N** | 1.52 | 0.22 | 2.83 | 0.099 | 0.0007 | 0.98 |
| **AG** | **27.57** | **<0.0001** | 1.26 | 0.27 | **37.26** | **<0.0001** |
| **BG** | 3.05 | 0.087 | 1.65 | 0.21 | 1.28 | 0.26 |
| **N×AG** | 0.76 | 0.39 | 0.18 | 0.68 | 0.61 | 0.44 |
| **N×BG** | 0.84 | 0.36 | 0.40 | 0.53 | 0.40 | 0.53 |
| **AG×BG** | **4.17** | **0.047** | **4.18** | **0.046** | 0.61 | 0.44 |
| **N×AG×BG** | 0.24 | 0.63 | 0.01 | 0.91 | 0.32 | 0.57 |
