# Supplementary material for: Aboveground insect herbivory increases plant competitive asymmetry, while belowground herbivory mitigates the effect
Source: PeerJ. 2016 Apr 4;4:e1867. doi: 10.7717/peerj.1867 (PMC4824911; doi:10.7717/peerj.1867)
Supplement: Table S4 [file peerj-04-1867-s004.docx]

|  |  | **Aggressivity** | |  | **Relative Yield** | | | |
| --- | --- | --- | --- | --- | --- | --- | --- | --- |
| **Treatment** |  |  | | **Treatment** | *Dactylis glomerata* | | *Festuca rubra* | |
|  |  | ***F-*value** | ***P-*value** |  | ***F-*value** | ***P-*value** | ***F-*value** | ***P-*value** |
| **N** |  | 0.69 | 0.41 | **N** | 2.51 | 0.12 | 0.0091 | 0.92 |
| **Herb** |  | **13.35** | **<0.0001** | **A** | **7.77** | **0.01** | **11.22** | **0.0015** |
|  | *0-A* |  | **0.037** | **B** | 3.51 | 0.067 | 0.56 | 0.46 |
|  | *B-AB* |  | 0.92 | **Harv** | 1.39 | 0.25 | 1.15 | 0.32 |
| **Harv** |  | **4.73** | **0.032** | **N×A** | 0.36 | 0.55 | 0.18 | 0.67 |
| **N**×**Herb** |  | 0.80 | 0.50 | **N×B** | 2.42 | 0.13 | 0.14 | 0.71 |
| **N**×**Harv** |  | 0.014 | 0.91 | **N×Harv** | 0.11 | 0.90 | 0.24 | 0.79 |
| **Herb**×**Harv** |  | 0.45 | 0.72 | **A×B** | 1.16 | 0.29 | 1.18 | 0.28 |
|  |  |  |  | **A×Harv** | 2.43 | 0.092 | 0.63 | 0.53 |
|  |  |  |  | **B×Harv** | 0.69 | 0.50 | 1.48 | 0.23 |
