# Supplementary material for: Aboveground insect herbivory increases plant competitive asymmetry, while belowground herbivory mitigates the effect
Source: PeerJ. 2016 Apr 4;4:e1867. doi: 10.7717/peerj.1867 (PMC4824911; doi:10.7717/peerj.1867)
Supplement: Table S5 — The treatments are denoted in the left-hand column. N0 = low nitrogen, N1 = high nitrogen. [file peerj-04-1867-s005.docx]

|  | *Dactylis glomerata* | | | | | | *Festuca rubra* | | | | | |
| --- | --- | --- | --- | --- | --- | --- | --- | --- | --- | --- | --- | --- |
|  | Monoculture | | | Mixture | | | Monoculture | | | Mixture | | |
| **Harvest** | **1** | **2** | **3** | **1** | **2** | **3** | **1** | **2** | **3** | **1** | **2** | **3** |
| **N_0_** |  |  |  |  |  |  |  |  |  |  |  |  |
| 0 | 1.64 | 0.49 | 0.22 | 0.77 | 0.22 | 0.08 | 1.43 | 0.44 | 0.16 | 0.75 | 0.23 | 0.09 |
| A | 1.36 | 0.43 | 0.19 | 0.76 | 0.25 | 0.11 | 0.67 | 0.27 | 0.16 | 0.19 | 0.09 | 0.04 |
| B | 1.60 | 0.39 | 0.19 | 0.90 | 0.20 | 0.09 | 1.34 | 0.34 | 0.13 | 0.61 | 0.17 | 0.07 |
| AB | 1.48 | 0.39 | 0.19 | 0.81 | 0.21 | 0.09 | 0.73 | 0.23 | 0.15 | 0.21 | 0.08 | 0.04 |
| **N_1_** |  |  |  |  |  |  |  |  |  |  |  |  |
| 0 | 1.70 | 0.50 | 0.24 | 0.84 | 0.25 | 0.09 | 1.57 | 0.48 | 0.16 | 0.88 | 0.23 | 0.09 |
| A | 1.64 | 0.52 | 0.20 | 1.02 | 0.28 | 0.11 | 0.99 | 0.27 | 0.17 | 0.29 | 0.08 | 0.05 |
| B | 1.62 | 0.50 | 0.20 | 1.03 | 0.26 | 0.10 | 1.43 | 0.42 | 0.15 | 0.66 | 0.19 | 0.07 |
| AB | 1.37 | 0.41 | 0.18 | 0.86 | 0.27 | 0.13 | 0.62 | 0.19 | 0.10 | 0.14 | 0.06 | 0.03 |
